# Tumor-derived exosomal circPSMA1 facilitates the tumorigenesis, metastasis, and migration in triple-negative breast cancer (TNBC) through miR-637/Akt1/β-catenin (cyclin D1) axis

**DOI:** 10.1038/s41419-021-03680-1

**Published:** 2021-04-28

**Authors:** Su-jin Yang, Dan-dan Wang, Shan-liang Zhong, Wen-quan Chen, Feng-liang Wang, Jian Zhang, Wen-xiu Xu, Di Xu, Qian Zhang, Jian Li, He-da Zhang, Jun-chen Hou, Ling Mao, Jin-hai Tang

**Affiliations:** 1 Department of General Surgery, the First Affiliated Hospital of Nanjing Medical University, Nanjing, 210029 P.R. China; 2 The Affiliated Cancer Hospital of Nanjing Medical University, Nanjing, Jiangsu 210009 P.R. China; 3 Center of Clinical Laboratory, Nanjing Medical University Affiliated Cancer Hospital Cancer Institute of Jiangsu Province, Nanjing, 210009 P.R. China; 4 Department of Obstetrics and Gynecology, Maternity and Child Health Care Hospital, Nanjing Medical University, Nanjing, 210009 P.R. China; 5 Department of Thyroid and Breast Surgery, the Affiliated Huai’an Hospital of Xuzhou Medical University, the Second People’s Hospital of Huai’an, Huai’an, 223002 P.R. China

**Keywords:** Breast cancer, Cancer microenvironment

## Abstract

Circular RNAs (circRNAs) are increasingly gaining importance and attention due to their diverse potential functions and their value as diagnostic biomarkers (disease specific). This study aims to explore the novel mechanisms by which exosome-contained circRNAs promote tumor development and metastasis in TNBC. We identified increased circRNA circPSMA1 in TNBC cells, their exosomes, and serum exosomes samples from TNBC patients. The overexpression of circPSMA1 promoted TNBC cell proliferation, migration, and metastasis both in vitro and in vivo. Moreover, we investigated the tumor-infiltrating immune cells (TICs) or stromal components in immune microenvironment (IME), and identified the significant differences in the immune cells between TNBC and non-TNBC samples. Mechanistically, circPSMA1 acted as a “miRNAs sponge” to absorb miR-637; miR-637 inhibited TNBC cell migration and metastasis by directly targeted Akt1, which recognized as a key immune-related gene and affected downstream genes β-catenin and cyclin D1. Subsequent co-culture experiments also demonstrated that exosomes from TNBC carrying large amounts of circPSMA1 could transmit migration and proliferation capacity to recipient cells. Kaplan–Meier plots showed that high expression of Akt1 and low expression of mir-637 are highly correlated with poor prognosis in patients with lymph node metastasis of TNBC. Collectively, all these results reveal that circPSMA1 functions as a tumor promoter through the circPSMA1/miR-637/Akt1-β-catenin (cyclin D1) regulatory axis, which can facilitate the tumorigenesis, metastasis, and immunosuppression of TNBC. Our research proposes a fresh perspective on novel potential biomarkers and immune treatment strategies for TNBC.

## Introduction

Aside from skin cancer, breast cancer is the most common cancer among women, and it is the second leading cause of cancer-related death among women in the United States^1^. Approximately 15% of all breast cancers are triple-negative breast cancer (TNBC), which is estrogen receptor (ER)-negative, progesterone receptor (PR)-negative, and human epidermal growth factor receptor 2 (HER2)-negative; TNBC generally exhibits a more aggressive clinical presentation^2^. Importantly, TNBC is identified more frequently in young women and has a high histological grade, lack of effective targeted therapeutic options, and poor prognosis. Therefore, the discovery of novel targeted molecular therapeutic approaches for TNBC patients is urgent.

Exosomes are small membrane-bound vesicles with sizes ranging from 30 to 100 nm^3^ that contain informational molecules such as proteins, mRNA, and lipids. Therefore, exosomes are well known by the “exosomal shuttle” that delivers oncogenic microRNAs (miRNAs), mRNAs, noncoding RNAs, and proteins to the recipient cells and tumor microenvironment^4,5^. Tumor-derived exosomes are reported to be involved in the development of tumor outgrowth and the formation of the premetastatic niche^6,7^. Recently, circular RNAs (circRNAs), a new type of endogenous noncoding RNA, have been identified to be enriched in exosomes, suggesting that they may be promising biomarkers for tumor diagnosis and prognosis^8,9^. CircRNAs are continuous, covalently closed RNAs that can be resistant to RNase R due to noncollinear joining of their 3′- and 5′-ends^10^. With the development of biotechnology and high-throughput sequencing technology, circRNAs are now regarded as abundant, diverse, and conserved molecules that act as pivotal regulators of many biological processes^11^. Although the biological functions of circRNAs are not completely understood, many of their functions, such as acting as miRNA sponges or “scaffolding” protein complexes to form RNA-protein complexes, have been demonstrated^12,13^. Some circRNAs were identified as tumor oncogenes in the progression of breast cancer^14–16^, however, few studies have found a relationship between circRNA and TNBC. Thus, the involvement of exosomal circRNAs in the mechanisms of TNBC development and metastasis should be further explored. In this study, we profiled the circRNAs in breast cancer cell lines and their exosomes by RNA sequencing and showed that circPSMA1 might act as an oncogenic gene in TNBC progression. Moreover, circPSMA1 could be a valuable marker and an independent prognostic factor for TNBC diagnosis and therapy.

## Results

### CircPSMA1 is upregulated in TNBC cells and their exosomes and in TNBC patients

**Fig. 1:**
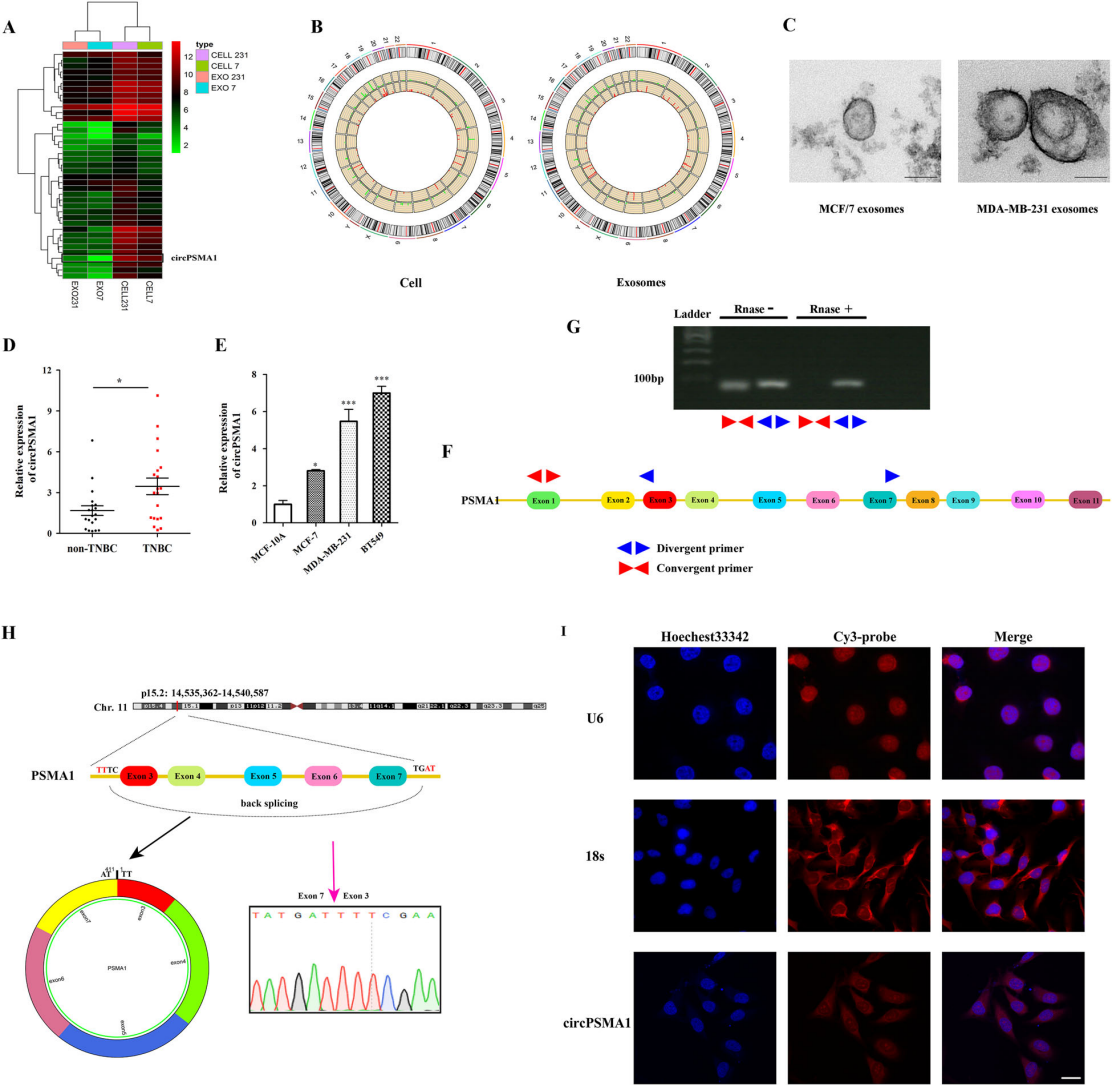
Expression profiles of circRNAs in breast cancer cells and their exosomes and the characteristics of circPSMA1. **A** The expression profiles of the top 39 differentially expressed circRNAs in MDA-MB-231 cells (TNBC cell line) and their exosomes compared with MCF-7 cells (non-TNBC cell line) and their exosomes. Pseudo-colors (green to red) indicate the expression levels from low to high. **B** The significantly differentially expressed circRNAs represent in Circos diagrams generated by Circos (http://circos.ca/) with the threshold criteria of fold change > 2.0 and *P* < 0.05. Red bars indicate upregulated circRNAs, while blue bars represent downregulated circRNAs. The height of the bar means the fold change of the expression level of a circRNA. **C** Representative images of exosomes from MDA-MB-231 cells and MCF-7 cells using a transmission electron microscope (TEM). Scale bar, 100 nm. **D** The expression level of circPSMA1 was verified in serum exosome samples from TNBC patients (*n* = 20) and non-TNBC patients (*n* = 20) by qRT-PCR analysis. **E** The expression level of circPSMA1 was verified in TNBC cell lines (BT-549 and MDA-MB-231), non-TNBC cell lines (MCF/7 and MCF-10A). **F** The schematic illustrates the regions of qRT-PCR primers for the genomic loci of PSMA1 gene and circPSMA1. Convergent primer for detecting linear PSMA1 and divergent primers for circPSMA1. **G** Gel electrophoresis analysis of RT-PCR for the existence of circPSMA1 in breast cancer cells. CircPSMA1 is only amplified by divergent primers in cDNA treated with RNase. **H** The genomic loci of PSMA1 gene and circPSMA1. Red arrow indicates the back-splicing region of PSMA1 exon 3 to exon 7 verified by Sanger sequencing. The circPSMA1 spliced mature sequence length is 411 nt. **I** The FISH assay shows the subcellular localization of circPSMA1. The circPSMA1 probe is labeled with Cy3 (red) and nuclei is stained with Hochest 33342 (blue) (magnification, ×200, Scale bar = 20 μm). All data were shown as mean ± SD at least three independent experiments, *p < 0.05, ***p < 0.001.

To understand the expression profiles of circRNAs in TNBC, we profiled the circRNAs in MDA-MB-231 cells (TNBC cell line) and their exosomes and compared them with the profiles in MCF-7 cells (non-TNBC cell line, which PR and ER were positive) and their exosomes^17^. The expression profiles of the top 39 differentially expressed circRNAs (both in cells and their exosomes) were identified via hierarchical clustering (Fig. 1A, Table S2), and the significantly differentially expressed circRNAs are represented in Circos diagrams generated by Circos (http://circos.ca/) (Fig. 1B). The characteristics and size of the exosomes were detected using a transmission electron microscope (TEM) (Fig. 1C). We found that the expression of circPSMA1 was significantly upregulated in MDA-MB-231 cells and their exosomes compared with MCF/7 cells and their exosomes. Then, the expression of circPSMA1 was detected in serum exosomes samples from TNBC patients (*n* = 20), non-TNBC patients (*n* = 20), TNBC cell lines (BT-549, MDA-MB-231), non-TNBC cell lines (MCF/7), and normal breast epithelial cells (MCF-10A) by qRT-PCR. As expected, the higher expression of circPSMA1 was found in serum exosome samples from TNBC patients (Fig. 1D) and TNBC cell lines (Fig. 1E) compared with that in non-TNBC patients and non-TNBC cell lines.

To confirm the existence of circPSMA1, convergent and divergent primers were designed to amplify linear PSMA1 and circPSMA1, respectively (Fig. 1F). The circPSMA1 was only amplified by divergent primers in cDNA treated with RNase (Fig. 1G). Then, we identified the back-splicing of circPSMA1 by Sanger sequencing (Fig. 1H). The FISH assay showed that circPSMA1 was predominantly located in the cytoplasm (Fig. 1I).

### CircPSMA1 promotes TNBC cells proliferation and migration and regulates cell cycle and apoptosis

**Fig. 2:**
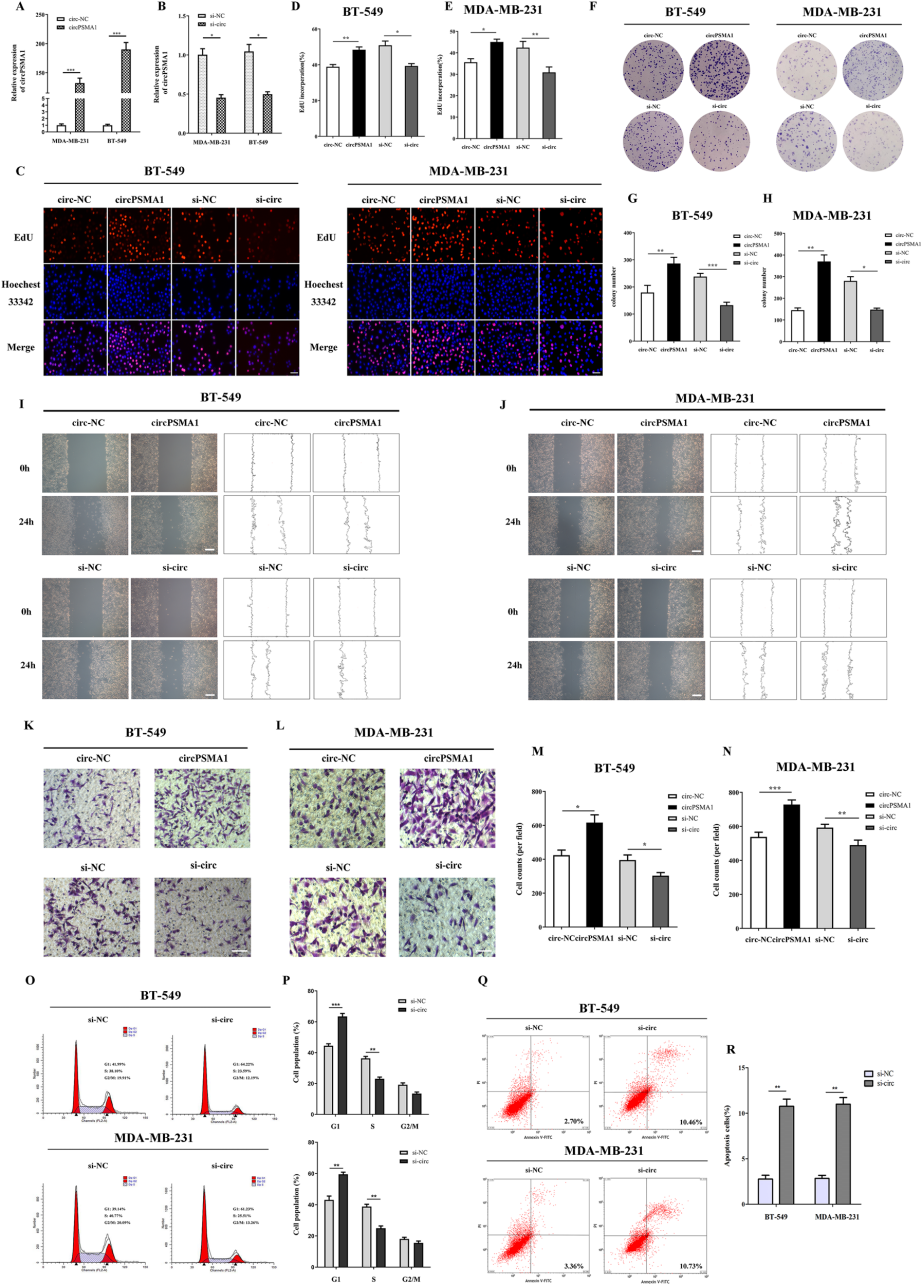
CircPSMA1 promotes TNBC cell proliferation and migration and regulates cell cycle and apoptosis. **A** The qRT-PCR analysis of circPSMA1 expression in TNBC cells transfected with circPSMA1 overexpression vector (circPSMA1) and negative control (circ-NC). **B** The qRT-PCR analysis of circPSMA1 expression in TNBC cells transfected with circPSMA1 siRNA vector (si-circ) and negative control (si-NC). **C**–**E** EdU assays were carried out in TNBC cells after transfection with circPSMA1 overexpression vector (circPSMA1) or circPSMA1 siRNA vector (si-circ) (magnification, ×100, Scale bar = 100 μm). **F**–**H** Colony formation assays were conducted to confirm the proliferative abilities of TNBC cells that transfected with these indicated vectors. **I**, **J** The wound healing assays were carried out to detect the migration abilities of TNBC cells after transfected with these indicated vectors (magnification, ×50, Scale bar, 200 μm). **K**–**N** Transwell assays were executed to detect the migration abilities of TNBC cells after transfected with these indicated vectors (magnification, ×100, Scale bar, 100 μm). **O**, **P** Cell cycle analysis showed that circPSMA1 could regulate TNBC cell cycle and apoptosis. **Q**, **R** The apoptotic rate of TNBC cells was analyzed by flow cytometry after downregulation of circPSMA1 expression. Data were shown as mean ± SD at least three independent experiments, *p < 0.05, **p < 0.01, ***p < 0.001.

To investigate the biological function of circPSMA1 in breast cancer cells, loss-of-function and gain-of-function assays were performed. The qRT-PCR results showed the circPSMA1 overexpression or knockdown efficiency of transfection with the circPSMA1 overexpression or RNAi vector (Fig. 2A, B); moreover, the analysis confirmed that the transfection had no influence on the expression of the parental PSMA1 gene (Fig. S2). EdU assays revealed that overexpression of circPSMA1 significantly increased the percentage of EdU-positive cells in BT-549 cells and MDA-MB-231 cells and that downregulation of circPSMA1 produced the opposite result (Fig. 2C–E). Similar to the effects seen in the EdU assays, colony formation assays also showed similar results (Fig. 2F–H). The wound healing assays and transwell assays displayed that the migration abilities of BT-549 and MDA-MB-231 cells were significantly promoted by circPSMA1 overexpression and markedly suppressed by circPSMA1 knockdown (Fig. 2I–N).

As it is well known that the cell cycle is a key component of neoplastic signaling pathways and abnormal cell cycle progression, we then explored the effect of circPSMA1 on cell cycle progression and apoptosis of breast cancer cells. As shown in Fig. 2O and Fig. 2P, more G1 phase cells and fewer S phase cells were observed in the circPSMA1 knockdown group compared with the control group, which implied that circPSMA1 could affect G1 arrest in breast cancer cells. In addition, the apoptotic rate was higher in circPSMA1 knockdown BT-549 and MDA-MB-231 cells than in control cells (Fig. 2Q, R). All these results confirmed that circPSMA1 promoted the proliferation, migration, and metastasis of TNBC cells and might regulate cell cycle and apoptosis.

### CircPSMA1 may function as a sponge of miR-637

**Fig. 3:**
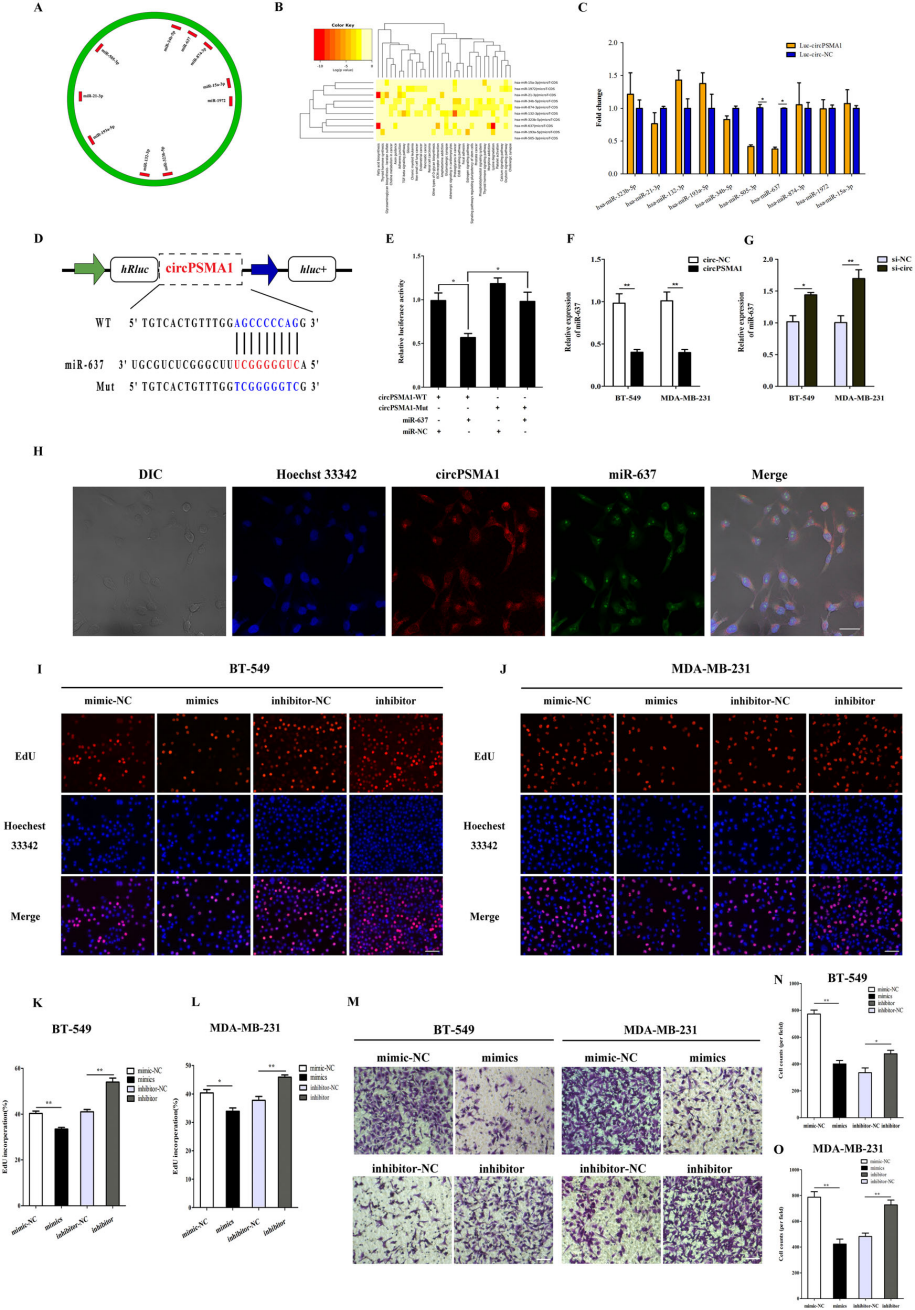
CircPSMA1 functions as a sponge of miR-637. **A** The 10 potential targeted miRNAs of circPSMA1 were predicted by three databases. The green circle means circPSMA1 and red rectangle indicates the targeted miRNAs. The image shows the binding sites of circRNA-miRNAs. **B** The signaling pathways of the 10 miRNAs were analyzed by DIANA-miRPath v3.0 (http://snf-515788.vm.okeanos.grnet.gr/). **C** The expression levels of these candidate miRNAs after cotransfected with luc-circPSMA1 (plasmid that overexpressed circPSMA1 and labeled with luciferase) or luc-circ-NC (plasmid that overexpressed circ-NC and labeled with luciferase) with candidate miRNA mimics. **D** Schematic illustration of circPSMA1-WT and circPSMA1-Mut luciferase reporter vectors and the miR-637 binding site on circPSMA1 predicted by miRanda. **E** The dual-luciferase reporter assay was carried out to validate whether miR-637 could directly bind to circPSMA1. After cotransfected circPSMA1-WT or circPSMA1-Mut luciferase reporter vectors with miR-637 mimic or NC respectively, the relative luciferase activities were detected by spectramax. **F**, **G** The qRT-PCR analysis detected the relative expression of miR-637 after overexpression or knockdown of circPSMA1. **H** FISH assay displayed the cellular location of circPSMA1 (red) and miR-637 (green) in MDA-MB-231 cells (magnification, ×200, scale bar, 50 μm). **I**, **L** EdU assays showed the percentage of EdU-positive cells in BT-549 and MDA-MB-231 cells after overexpression or knockdown of miR-637 by mimic and inhibitor (magnification, ×100, Scale bar, 100 μm). **M**–**O** Transwell assays detected the migration abilities of TNBC cells after overexpression or knockdown of miR-637 by transfected with miR-637 mimic, miR-637 inhibitor or their NC (mimic-NC and inhibitor-NC) (magnification, ×100, Scale bar, 100 μm). Data were shown as mean ± SD at least three independent experiments, *p < 0.05, **p < 0.01.

Increasing evidence reports that circRNAs act as “miRNA sponges” in tumor progression and metastasis^18–20^. To elucidate the underlying mechanism of circPSMA1, we predicted 10 potential target miRNAs of circPSMA1 by cross-analyzing three databases: RNAhybrid, TargetScan, and miRanda (Fig. 3A). The signaling pathways of the 10 miRNAs were investigated by DIANA-miRPath v3.0 (Fig. 3B). Importantly, certain miRNAs were related to some breast cancer-related pathways, such as ECM-receptor interaction, focal adhesion, and estrogen and TGF-beta signaling pathways.

Next, we cotransfected the luc-circPSMA1 plasmid or NC vector with candidate miRNA mimics to preliminarily screen the potential miRNAs that interact with circPSMA1. As shown in Fig. 3C, the specific decrease of miR-505 and miR-637 were detected, with levels significantly lower than those of the control group. To confirm the absorption of miR-637 and circPSMA1, we performed a dual-luciferase reporter assay (Fig. 3D). The results indicated that miR-637 mimics could significantly decrease the luciferase activity of the circPSMA1-WT group but not the mutant group (Fig. 3E), suggesting a direct interaction between miR-637 and circPSMA1. In addition, overexpression of circPSMA1 led to markedly decreased expression of miR-637, while silencing of circPSMA1 significantly increased the expression of miR-637 in BT-549 and MDA-MB-231 cells (Fig. 3F, G). Moreover, the FISH assay confirmed the colocalization of circPSMA1 and miR-637 in the cytoplasm (Fig. 3H).

To further explore the mechanism of miR-637 in TNBC proliferation and metastasis, we overexpressed and downregulated the level of miR-637 in BT-549 and MDA-MB-231 cells. The result of EdU assays showed that the overexpression of miR-637 significantly decreased the percentage of EdU-positive cells in BT-549 and MDA-MB-231 cells, while downregulation of miR-637 showed the opposite effect (Fig. 3I–L). Moreover, transwell assays revealed that the migration abilities of BT-549 and MDA-MB-231 cells were significantly promoted by downregulated miR-637 expression and that these abilities were markedly suppressed by increased expression of miR-637 (Fig. 3M–O). Therefore, miR-637 may function as an antineoplastic factor to inhibit TNBC cell proliferation, migration and metastasis.

### TICs and TME scores were associated with the survival and clinicopathological characteristics of TNBC patients and immune-related target genes of miR-637

**Fig. 4:**
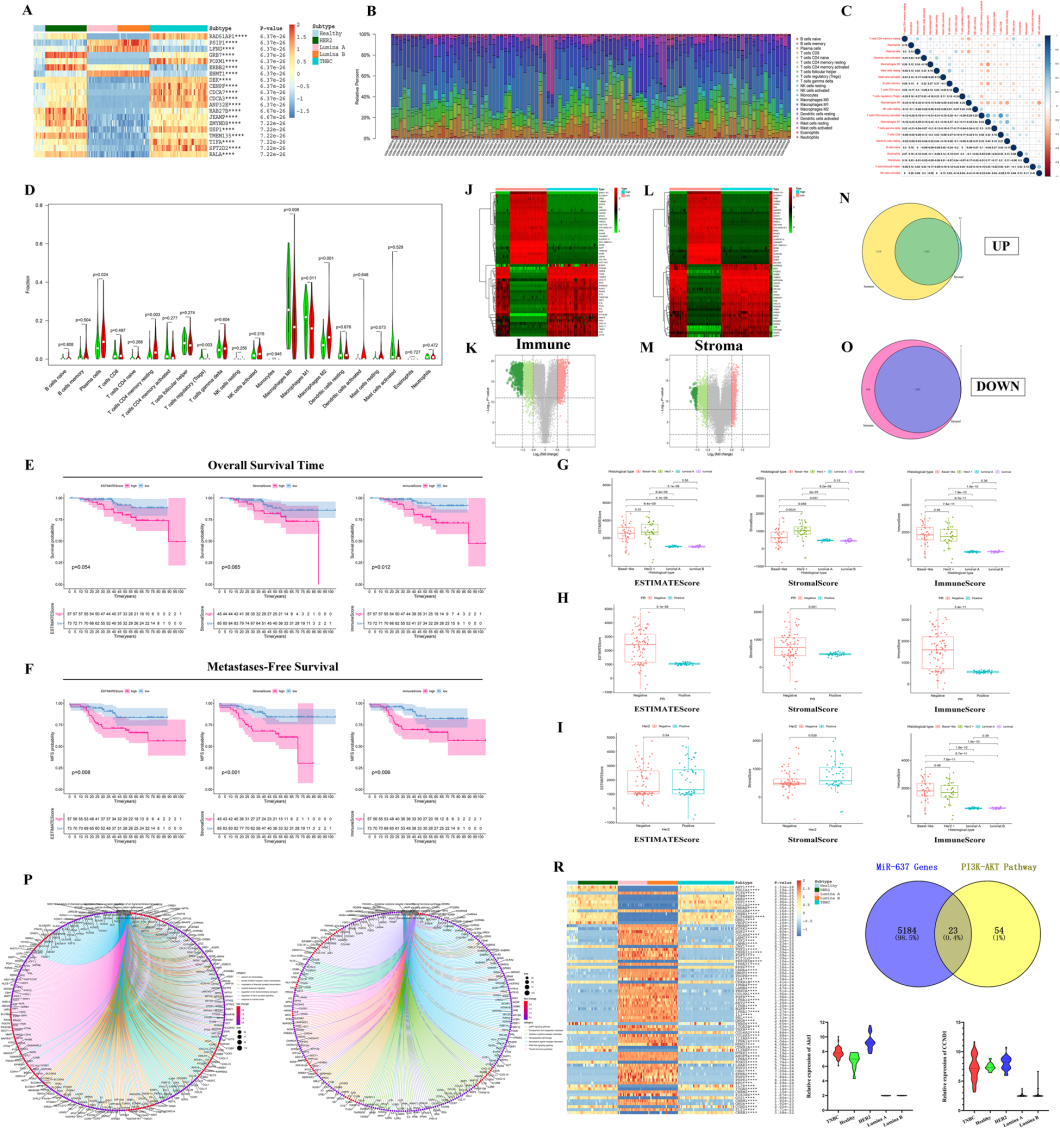
TICs, TME scores, and PI3K-Akt signaling pathway were associated with the survival and clinicopathological characteristics of TNBC patients. **A** The top 20 DEGs in five different types of breast cancer and normal breast tissue samples (GSE65194), ****P-value < 0.0001. [TNBC (*n* = 55), Her2 (*n* = 39), Luminal B (*n* = 30), Luminal A (*n* = 29), Healthy (*n* = 11)]. **B** The barplot showing the different proportions of 22 TICs in different types of breast cancer samples (*n* = 103). **C** The correlations between 22 different kinds of TICs. The value in each small box represents the *P* value of correlation between the two kinds of TICs. The chroma of each small color box represents the corresponding correlation value between the two kinds of TICs. Pearson coefficient is used for the significance test. **D** The violin plot showed the significantly differentially expression of immune cells between TNBC (*n* = 55) and non-TNBC (*n* = 48) samples. **E**, **F** Kaplan–Meier survival analysis depending on overall survival (OS) (**E**) or metastases-free survival (MFS) (**F**) was performed for breast cancer patients with high or low ESTIMATEScore, StromalScore, and ImmuneScore, respectively. **G**–**I** Distribution of ESTIMATEScore, StromalScore, and ImmuneScore in histological type (**G**), PR (**H**), Her2 (**I**). **J**, **K** The heatmap (**J**) and volcano (**K**) for 3378 DEGs. The patients were grouped according to their high and low ImmuneScore. **L**, **M** The heatmap (**L**) and volcano (**M**) for 3378 DEGs. The patients were grouped according to their high and low StromalScore. The DEGs were calculated by Wilcoxon rank sum test. The results with the criterion of logFC > 0.8, *P* < 0.01, and false discovery rate (FDR) < 0.01 as the significance threshold. **N**–**O** The venn diagram showed 1153 upregulated DEGs (**N**) and 2591 downregulated DEGs (**O**) both in data regarding ImmuneScore or StromalScore. **P**–**Q** GO and KEGG enrichment analysis for 3744 DEGs displayed the enrichment of immune-related GO terms (**P**) and signaling pathways (**Q**), with *p* and *q* value < 0.05 as the significance threshold. **R** The heatmap showed that the 77 genes enriched in the PI3K-Akt signaling pathway have different expression levels in different subtypes of BCa, ****p < 0.0001. **S** The Venn diagram showed the 23 target genes through the cross-analysis of genes from PI3K-Akt signaling pathway and target genes of miR-637. **T**, **U** The expression levels of Akt1 (**T**) and CCND1 (cyclinD1) (**U**) between different types of breast cancer.

Recently, some studies have shown that the new mechanism of TNBC tumor metastasis may be related to tumor-infiltrating immune cells (TICs) and stromal components in the microenvironment of tumor and its pre-metastasis, and exosomes also play a very critical role in this process by delivering some noncoding RNAs. The heatmap shows the first 20 DEGs in five different types of samples (Fig. 4A). The relative percent of different TICs and the correlation between TICs were shown in Fig. 4B and C. The violin plot showed that the significantly differentially expression of immune cells in TNBC (*n* = 55) and non-TNBC (*n* = 48) tumors (Fig. 4D), such as CD4 memory resting T cells, regulatory T cells (Tregs), and macrophages (M0, M1, M2).

Kaplan–Meier survival analysis was used for different kinds of scores depending on OS time and the metastasis time, respectively. There were significant differences in the OS of BCa patients between the high and low ImmuneScore group (*p* = 0.012) (Fig. 4E). Moreover, metastases-free survival (MFS) was highly correlated with the ESTIMATEScore (*p* = 0.008), StromalScore (*p* = 0.0009), and ImmuneScore (*p* = 0.006) and in TME, suggesting that BCa patients with higher scores are more likely to occur metastases (Fig. 4F).

We further investigated the relationship between TME score and clinicopathological features. Compared with luminal A and B subtypes, TNBC (Basal-like) and Her2+ subtypes showed significantly higher expression in TME score, and Her2+ subtype showed a significantly higher score in StromalScore than other subtypes (Fig. 4G). The PR and Her-2 positive BCa samples also showed significantly higher TME scores than that of PR negative samples (Fig. 4H, I).

A total of 3378 DEGs and 5889 DEGs obtained from StromalScore and ImmuneScore were used to identify the genes of immune and stromal components associated with TME remodeling (Fig. 4J–O). GO and KEGG enrichment analysis displayed the enrichment of immune-related GO terms (Fig. 4P) and pathways (Fig. 4Q). Interestingly, 77 DEGs were found to be enriched in the PI3K-Akt signaling pathway (Fig. 4R) and identified 23 target genes of miR-637 by cross-analysis (Fig. 4S). The results of comparison analysis showed that Akt1 and CCND1 (cyclinD1) were significantly different in different types of breast cancer (Fig. 4T, U, Tables S5, S6). According to previous studies, we chose Akt1 as the target gene of miR-637 for the next study.

### MiR-637 inhibits TNBC cells proliferation, migration, and metastasis by directly targeted Akt1

**Fig. 5:**
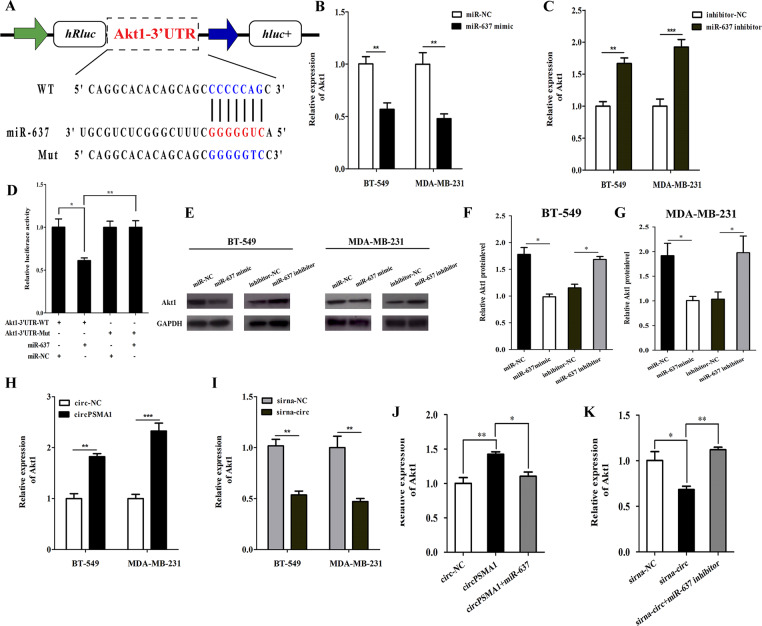
MiR-637 inhibits TNBC cell proliferation, migration, and metastasis by directly targeted Akt1. **A** Schematic illustration of Akt1-3′UTR-WT and Akt1-3′UTR-Mut luciferase reporter vectors and the miR-637 binding site on Akt1 that predicted by TargetScan (http://www.targetscan.org/vert_72/). **B** The dual-luciferase reporter assay was carried out to validate whether miR-637 could directly bind to the 3′-UTR of Akt1. After cotransfected Akt1-3′UTR-WT and Akt1-3′UTR-Mut luciferase reporter vectors with miR-637 mimic or NC respectively, the relative luciferase activities were detected by spectramax. **C**–**G** The relative expression of Akt1 after overexpression or knockdown of miR-637 by transfected with miR-637 mimic, miR-637 inhibitor, mimic-NC or inhibitor-NC was detected by qRT-PCR and western blot, respectively. **H**–**I** The relative expression of Akt1 in TNBC cells after overexpression or knockdown of circPSMA1 was detected by qRT-PCR. **J**–**K** The relative expression of Akt1 after cotransfecting the indicated vectors with miR-637 mimic, miR-637 inhibitor, mimic-NC or inhibitor-NC. Data were shown as mean ± SD at least three independent experiments, *p < 0.05, **p < 0.01, ***p < 0.001.

TargetScan was used to confirm that Akt1 shares the same MRE with miR-637. The dual-luciferase reporter assay was carried out to validate whether miR-637 could directly bind to the 3′-UTR of Akt1 compared with control groups (Fig. 5A). The results showed that overexpression of miR-637 by miR-637 mimics dramatically decreased the luciferase activity of the reporter of the wild-type (WT) 3′-UTR of Akt1 (Fig. 5D). Furthermore, we found that the expression level of Akt1 was regulated by increasing and decreasing the level of miR-637 in TNBC cells (Fig. 5B, C). Consistently, the Akt1 protein expression levels were accordingly altered by miR-637 mimics and inhibitors in TNBC cells (Fig. 5E–G). As expected, the expression of Akt1 could be regulated by circPSMA1; overexpression of circPSMA1 increased the expression of Akt1 and knockdown of circPSMA1 significantly decreased the expressions of Akt1 (Fig. 5H, I). In addition, enhancement of Akt1 expression caused by circPSMA1 overexpression was abolished by ectopic expression of miR-637, while inhibition of Akt1 expression due to knockdown of circPSMA1 was partly eliminated by reducing the level of miR-637 (Fig. 5J, K). All these experiments suggested that miR-637 directly targets Akt1 and regulates the expression level of Akt1 in TNBC cells.

### CircPSMA1 promotes TNBC cell proliferation and migration through the circPSMA1/miR-637/Akt1 axis in vitro

**Fig. 6:**
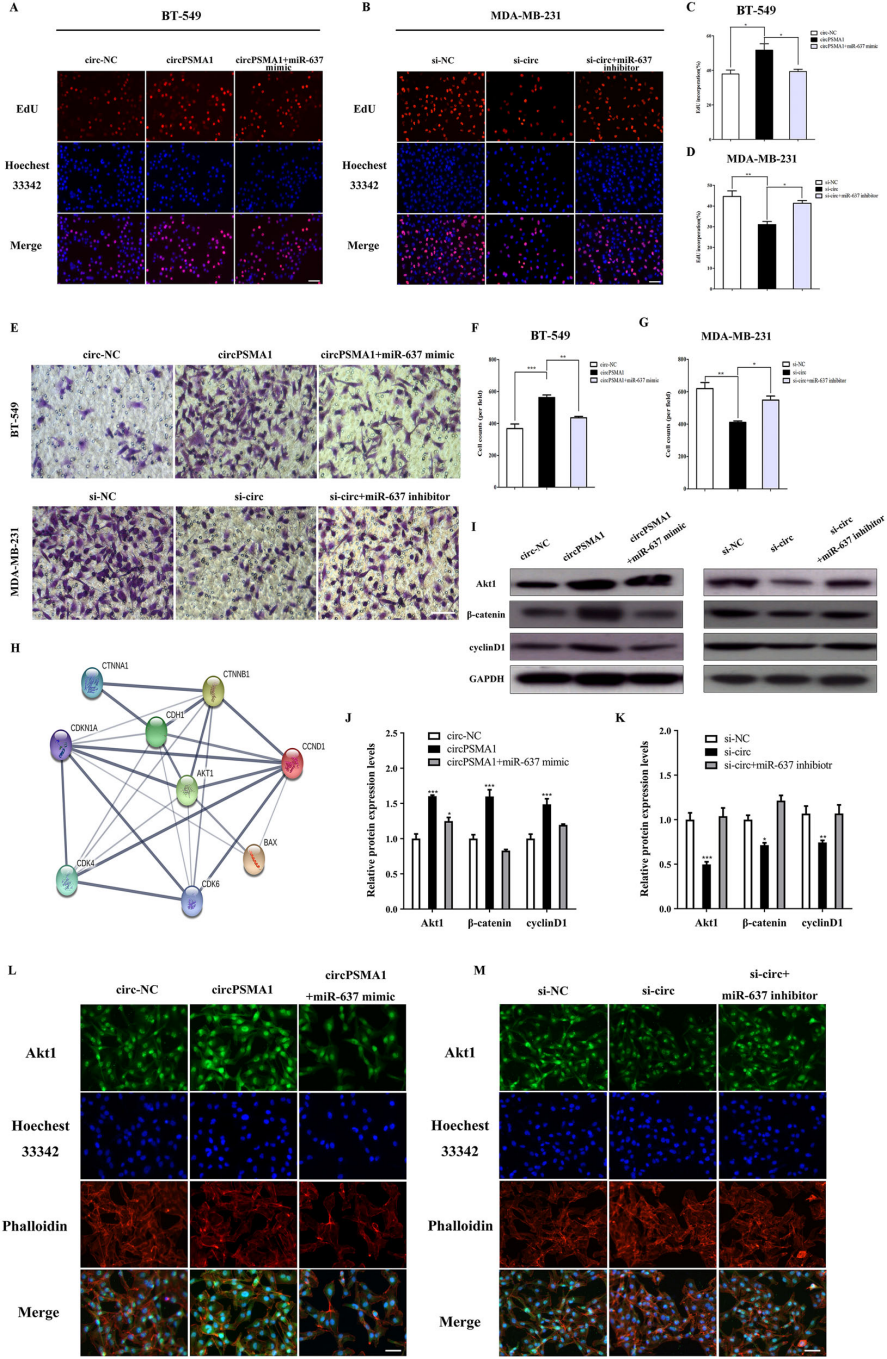
CircPSMA1 promotes TNBC cell proliferation and migration through the circPSMA1/miR-637/Akt1 axis in vitro. **A**–**D** The cell proliferation of TNBC cells after cotransfecting the indicated vectors with miR-637 mimic or miR-637 inhibitor was detected by EdU assays (magnification, ×100, scale bar, 100 μm). **E**–**G** The migration abilities of TNBC cells after cotransfecting the indicated vectors with miR-637 mimic or miR-637 inhibitor were detected by transwell assays (magnification, ×100, Scale bar, 100 μm). **H** STRING database (https://string-db.org/) was used to confirm the relationship of β-catenin (CTNNB1) and cyclin D1 (CCND1) with Akt1. **I**–**K** The relative expressions of Akt1, β-catenin and cyclin D1 after cotransfecting the indicated vectors with miR-637 mimic or miR-637 inhibitor were determined by western blot. **L**, **M** Relative protein levels of Akt1 after cotransfecting the indicated vectors with miR-637 mimic or miR-637 inhibitor in TNBC cells were assessed by immunofluorescence (IF) (magnification, ×100, Scale bar, 50 μm). Data were shown as mean ± SD, *p < 0.05, **p < 0.01, ***p < 0.001.

To further explore the mechanism of circPSMA1, several rescue experiments were conducted to illustrate how the circPSMA1/miR-637/Akt1 axis promotes TNBC cell proliferation and migration. Functionally, the EdU assay revealed that overexpression of circPSMA1 markedly enhanced the proliferative abilities of BT-549 and MDA-MB-231 cells, and this enhancement could be blocked by a miR-637 mimic; knockdown of circPSMA1 significantly suppressed the proliferative abilities of the cells, and the suppression could be partly rescued by miR-637 inhibitors (Fig. 6A–D). In addition, transwell assays showed similar effects on the invasive capacity of the cells (Fig. 6E–G).

Previous studies reported that Akt1 might have some target proteins that are involved in cell proliferation and migration, such as β-catenin (CTNNB1) and cyclin D1 (CCND1)^21–24^. We used the STRING database (https://string-db.org/) to confirm the relationship of these proteins with Akt1 (Fig. 6H). As expected, western blot and IF staining assay suggested that overexpression of circPSMA1 enhanced the protein level of Akt1, meanwhile, increased the protein level of cyclin D1 and β-catenin; while knockdown of circPSMA1 remarkably decreased the proteins levels of Akt1, cyclin D1, and β-catenin. More importantly, these changes caused by overexpression or silencing of circPSMA1 could be partly reversed by miR-637 mimic or inhibitor, respectively (Fig. 6I–L). In summary, these data demonstrated that circPSMA1 might serve as a ceRNA that acts as a sponge for miR-637 to regulate miR-637 activity and Akt1 expression.

### Exosomes derived from TNBC cells can promote cells proliferation and migration through modulate the expression of circPSMA1/Akt1/β-catenin

**Fig. 7:**
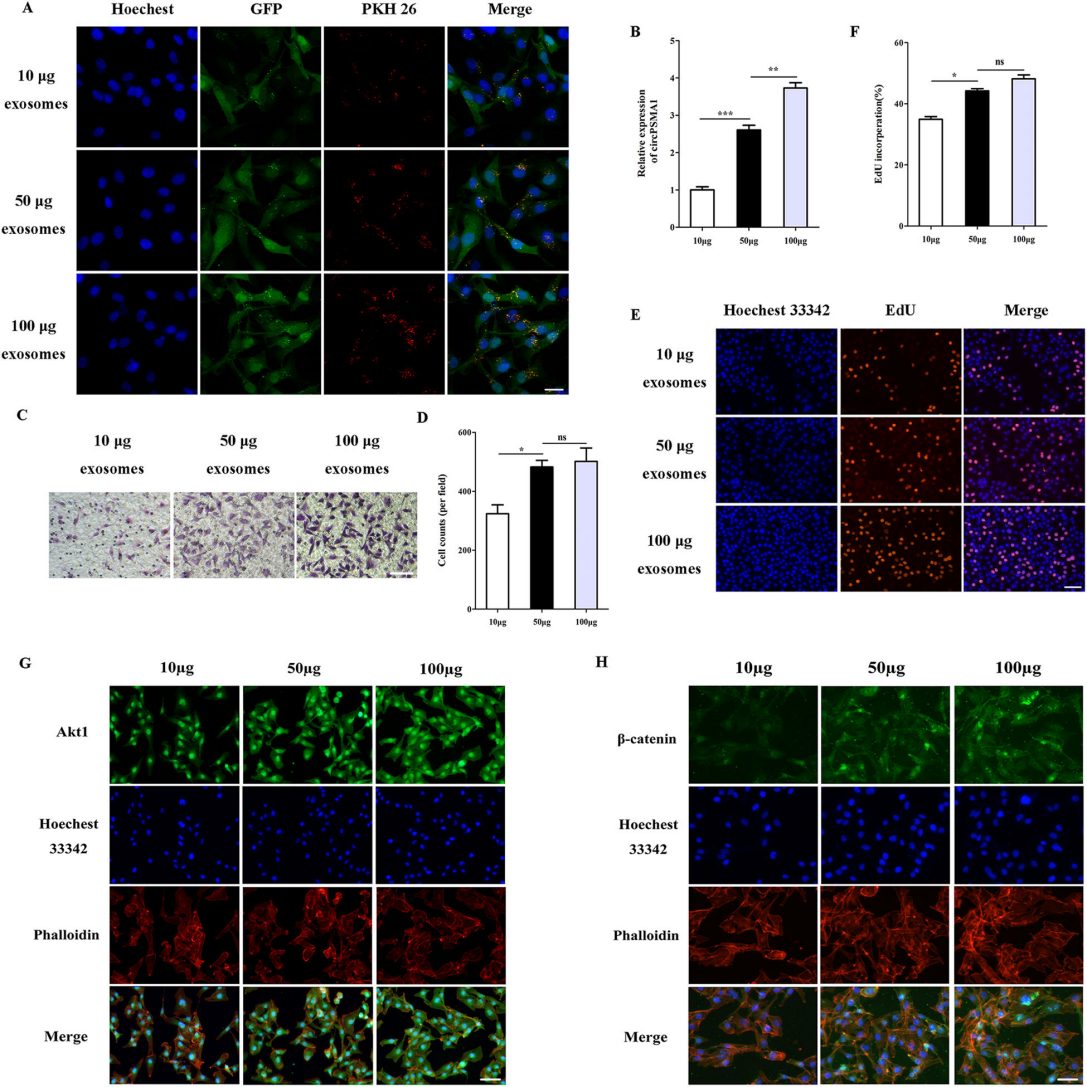
Exosomes derived from TNBC cells can promote cell proliferation and migration through modulate the expression of circPSMA1/ Akt1/β-catenin. **A** The exosome absorption by MDA-MB-231 cells was visualized by IF (magnification, ×100, Scale bar, 50 μm). **B** The circPSMA1 expression level of MDA-MB-231 cells treated with different concentrations of exosomes. **C**–**D** The migration abilities of MDA-MB-231 cells after treated with different concentrations of exosomes were detected by transwell assays (magnification, ×100, Scale bar, 100 μm). **E**–**F** The proliferative abilities of MDA-MB-231 cells after treated with different concentrations of exosomes were shown by EdU assays (magnification, ×100, Scale bar, 100 μm). **B**–**H** Protein levels of Akt1 and β-catenin after treated with different concentrations of exosomes in MDA-MB-231 cells were assessed by IF (magnification, ×100, Scale bar, 50 μm). Data were shown as mean ± SD, *p < 0.05, **p < 0.01, ***p < 0.001.

Exosomes are known to play vital functions on cell-cell communication and thus alter the physiological activities of the recipient cells through the bioactive factors contained in exosomes, including circRNAs. To study the effect of exosomes derived from TNBC cells in the transfer of migration ability, different concentrations of exosomes (PKH26 labeled) from BT-549 cells were added to the culture medium of MDA-MB-231 cells (Fig. 7A). The fluorescence images showed the fluorescence intensity was related to the amount of exosome uptake, moreover, the circPSMA1 expression level was also related to the amount of exosome uptake (Fig. 7B). The results of the transwell showed that MDA-MB-231 cells treated with different concentrations of exosomes displayed the enhancement of migration of tumor cells (*P* < 0.01) (Fig. 7C, D). Moreover, MDA-MB-231 cells treated with higher concentrations of exosomes had higher proliferative abilities than those treated with lower concentrations of exosomes (*P* < 0.05) (Fig. 7E, F). Next, IF assay showed that MDA-MB-231 cells treated with more exosomes could increase the protein level of Akt1 and β-catenin (Fig. 7G, H).

### CircPSMA1 facilitates tumorigenesis, migration, and metastasis of TNBC cells in vivo

**Fig. 8:**
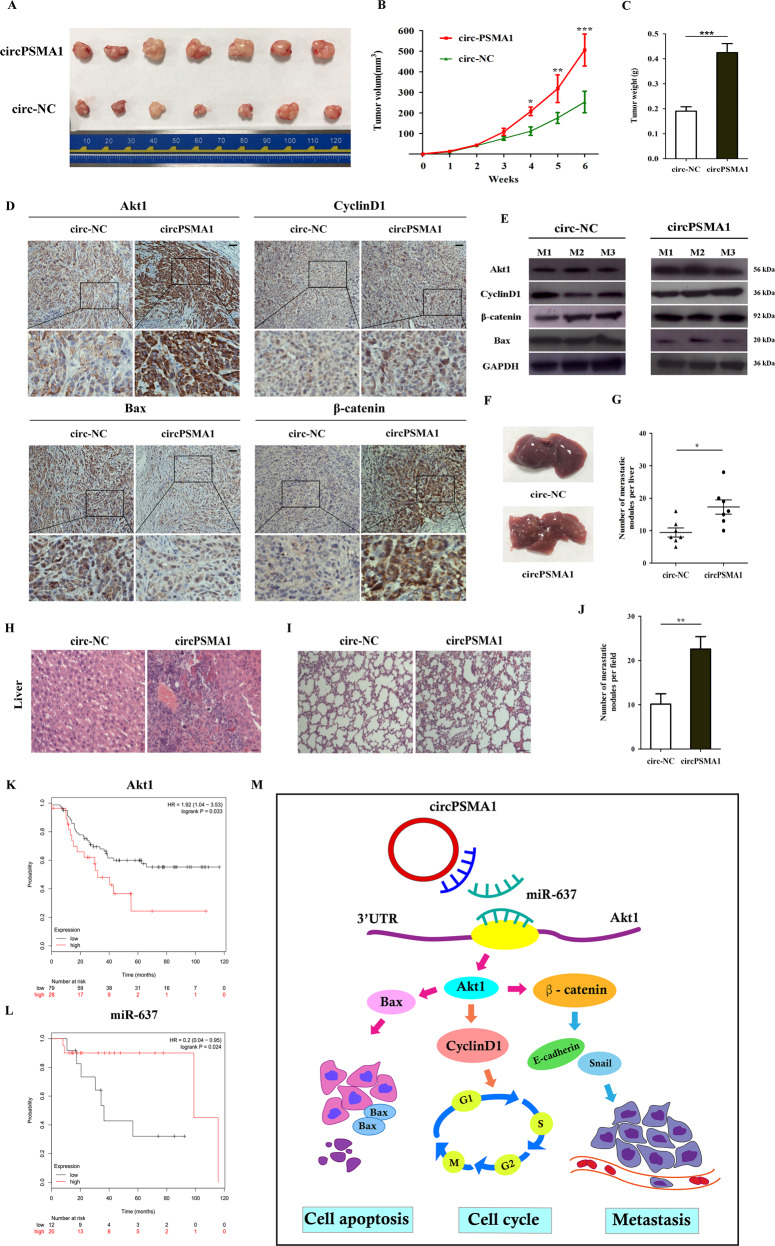
CircPSMA1 facilitates tumorigenesis, migration, and metastasis of TNBC cells in vivo. **A** Representative images of tumors of circPSMA1-overexpressing group (*n* = 7) or NC groups (*n* = 7). **B** Growth curves of tumors were measured weekly. **C** Tumor weight. **D** Immunohistochemistry (IHC) staining showed the protein levels of Akt1 and Akt1-related molecules (β-catenin, cyclin D1, and Bax) (magnification, ×200, Scale bar, 100 μm). **E** The protein level of Akt1 and Akt1-related molecules were detected by western blot. **F**–**H** The appearance and HE stain images of livers in two groups (magnification, ×100, Scale bar, 100 μm). **I**–**J** The HE stain images of lungs in two groups (magnification, ×50, Scale bar, 100 μm). **K**–**L** Kaplan–Meier survival analysis of overall survival (OS) in TNBC patients with lymphatic metastases based on TCGA data according to the Akt1 (*n* = 107) or miR-637 expression (*n* = 32). **M** The illustration of how circPSMA1/miR-637/Akt1 axis promoted TNBC cell proliferation, migration, and metastasis. CircPSMA1 functions as a sponge of miR-637 and downregulates miR-637 expression, and then increases Akt1 expression by directly binding miR-637 with the 3′-UTR of Akt1. Akt1 can upregulate cyclin D1 expression and lead to G1/S phase cell cycle arrest; Akt1 can upregulate β-catenin expression and cause to tumor migration and metastasis﻿; Akt1 can downregulate Bax expression and thereby inhibiting cell apoptosis. Therefore, circPSMA1 promoted TNBC cell proliferation, migration and metastasis through circPSMA1/miR-637/Akt1/β-catenin (cyclin D1) pathway.

To further study the effects of circPSMA1 on the development and metastasis of TNBC cells in vivo, circPSMA1 was stably overexpressed in MDA-MB-231 cells via transduction with lentivirus. CircPSMA1-overexpressing MDA-MB-231 cells (*n* = 7) and NC cells (*n* = 7) were gently injected into the mammary fat pad of female BALB/c nude mice, respectively. The volumes and weights of the tumors derived from cells overexpressing circPSMA1 were significantly larger and heavier than those of the tumors derived from NC cells (Fig. 8A–C). Then, all tumors were subjected to IHC staining to determine the effects of circPSMA1 on Akt1 and downstream proteins, such as cyclin D1, β-catenin, and Bax. The expressions of Akt1, cyclin D1, and β-catenin were significantly upregulated and the expression of Bax was notably downregulated in the circPSMA1-overexpressing group compared to the NC group (Fig. 8D). Western blot analysis further confirmed that the upregulation of circPSMA1 might increase the expression of Akt1, cyclin D1, and β-catenin but suppress the expression of Bax in xenograft tumor tissues (Fig. 8E). In addition, compared with the NC conditions, circPSMA1 overexpression promoted notable metastatic ability in the livers and lungs of mice (Fig. 8F–J). To explore the relationship between circPSMA1/miR-637/Akt1 pathway and clinical outcome of TNBC patients, we performed the Kaplan–Meier analysis to determine the patient’s actuarial OS. Kaplan–Meier plots showed that high Akt1 expression (log-rank test, *p* = 0.033) and low miR-637 expression (log-rank test, *p* = 0.024) was closely associated with poor outcome in TNBC patients with lymphatic metastases, whose data were obtained from the TCGA database (Fig. 8K, L). Taken together, these results suggest that circPSMA1 facilitates the tumorigenesis, migration, and metastasis of TNBC cells in vivo.

## Discussion

In recent years, numerous circRNAs have been identified, which are formed by head-to-tail splicing of exons; these molecules have unrecognized regulatory potential^25^. Moreover, some reports have shown that circRNAs participate in the progression of TNBC by absorbing targeted miRNAs and thus affecting the expression of downstream genes^26,27^. Previous studies revealed that certain circRNAs are cell-specific or tissue-specific^28^, circRNAs are recognized as promising novel biomarkers for the diagnosis and prognosis of some diseases, especially tumors^29^. Exosomes, a type of lipid bilayer membrane-bound vesicle containing RNA (mRNA, miRNA, and noncoding RNAs), proteins, and lipids, are also recognized as promising biomarkers for the diagnosis and prognosis of many cancers^30,31^. Exosomes participate in cell-to-cell communication by transferring numerous circRNAs, which contribute to tumor development and metastasis^32,33^. Our study systematically analyzes the mechanisms of exosome-contained circRNAs in TNBC and their potential clinical application in the treatment and prognosis of TNBC.

We first applied the RNA-seq profile to determine the circRNAs that are differentially expressed in TNBC cells and non-TNBC cells and their exosomes and identified circPSMA1, an upregulated circRNA, in both serum exosomes from TNBC patients and TNBC cell lines. Further, circPSMA1 facilitated the proliferation, migration, and metastasis of TNBC cells, as confirmed by functional experiments. According to the ceRNA hypothesis, some circRNAs, as highly conserved RNAs, might contain many miRNA-binding sites and function as “miRNA sponges” to regulate downstream gene expression by blocking the binding of miRNAs with 3′-UTR of their target genes^34,35^.

Recently, it has been found that TME plays important role in the initiation and development of cancer, especially in breast cancer^36,37^. Besides to cancer cell biology, the immune microenvironment (IME) that surrounding cancer cells, has also been found to affect tumorgenesis and metastasis of breast cancer. Therefore, it is useful to calculate the quality and quantity of immune components (immune stroma and cells) in the IME, as it may help to identify which patients can benefit from the following molecular therapies and predict the possible therapeutic targets. In our study, we found that compared with the non-TNBC samples, M0 and M1 macrophages were significantly reduced and M2 macrophages were significantly increased in TNBC samples. Therefore, we believe that there may be a huge imbalance in the immune cell microenvironment in the tumor tissues of TNBC, which leads to the inability to kill tumor cells correctly and leads to tumor immunosuppression. Not all immune cells are capable of inhibiting tumor development and metastasis, on the contrary, some immune cells act as pro-cancer factors. Increased regulatory T cells (Tregs) and CD4 memory T cells may contribute to the immunosuppressive IME and suggest a worse prognosis for BCa patients^38,39^. Similarly, some studies have reported that certain breast tumors exhibited disordered IME which infiltrated by large populations of M2 macrophages and regulatory T cells (Tregs)^40,41^.

It is well known that PI3K-Akt signaling pathway is not only closely related to the development and tumor metastasis of TNBC, but also recent studies have shown that the inhibition of PI3K-Akt signaling pathway mediated by PD-1/PD-L1 pathway could directly affect the proliferation and activation of T cells, thus inhibiting the process of anti-tumor immunity^42,43^. Previous studies have reported that miR-637 inhibits tumorigenesis, migration, and metastasis in some other cancers. Zhang et al. reported that miR-637 inhibits tumorigenesis in hepatocellular carcinoma by targeting signal transducer and activator of transcription 3 (Stat3)^44^. Similarly, Wang et al.^45^ also reported that miR-637 suppressed the proliferation and migration of hepatoma cells by targeted degradation of Akt1. It is worth mentioning that mining the data from the TargetScan database, the tumor suppressor miR-637 has other Akt isoforms as gene targets like Akt2 and Akt3 in addition to Akt1. Recent studies have shown that Akt2 and Akt3 may also be involved in the development and metastasis of TNBC^46^. Toker et al. found that Akt3 silencing markedly inhibited the growth of TNBC lines in mouse xenograft models and had no effect on invasion and metastasis^47^. Akt3-derived circRNA was also identified to enhance TNBC chemo-sensitivity through PI3K/Akt/mTOR signaling^48^. Akt3 did not appear in PI3K-Akt signaling pathway, and it was confirmed that Akt3 did not affect the invasion and metastasis of TNBC. Therefore, we chose Akt1 as the downstream target molecule of miR-637 for further research. Consistent with this, our results showed that miR-637 could inhibit TNBC migration and metastasis by directly targeting Akt1, thereby activating Akt1-related genes.

Interestingly, some reports also have suggested that Akt1 might target some proteins, such as β-catenin and cyclin D1, to alter the progression of cancer through regulating cell proliferation and migration^21–24^. Pestell et al. found that Akt1 governs breast cancer progression: Akt1 deficiency was verified to delay tumor growth and reduce lung metastases; moreover, Akt1-deficient mammary could reduce mammary epithelial tumor cell size and proliferative capacity by reducing cyclin D1 and p27 (KIP1) expression^49^. Cyclin D1 plays a critical role in regulating cell cycle progression; in addition, overexpression of cyclin D1 is an independent prognostic indicator in estrogen receptor (ER)-negative breast cancer patients^50^. Recently, Wnt/β-catenin signaling activation was found to play an important role in TNBC development and progression and is related to a poor clinical outcome^51^. In addition, Akt1-related pathways could affect the expression of EMT markers such as Snail1, E-cadherin, and N-cadherin, which promote tumor metastasis^22,52^. In addition, cyclin D1 and β-catenin were also predicted to be indicators of prognosis in breast cancer patients^50,51^. We confirmed that the high expression of Akt1 and the low expression of miR-637 were connected to poor prognosis in TNBC patients with metastases. Recent studies also reported that downregulation of miR-637 is an unfavorable prognostic biomarker for glioma^53^; moreover, high levels of pAkt1 were associated with reduced disease-free survival (DFS) and OS in breast cancer patients^54^.

In summary, this is the first study to explore the expression profile and mechanism of exosome-contained circRNAs related to TME and IME in TNBC. Our study integrally demonstrates that the circPSMA1/miR-637/Akt1-β-catenin (cyclin D1) regulatory circuit can facilitate the tumorigenesis, metastasis, and immunosuppression of TNBC via the activation of the Akt1-β-catenin (cyclin D1) signaling pathway (Fig. 8M). More importantly, these targets may be new potential prognostic biomarkers and immune treatment strategies to prevent the development and metastasis of TNBC.

## Materials and methods

### Samples collection

Forty serum samples from TNBC patients (*n* = 20) and non-TNBC patients (*n* = 20) were recruited from the Affiliated Cancer Hospital of Nanjing Medical University from 2017 to 2019. Eighth edition of American Joint Committee on Cancer was used to diagnose patients. The clinical characteristics of all patients were shown in additional file 1: Table S1. Fifteen milliliters of blood sample from each patient was collected into a sterile coagulation tube (Vacutainer System, BD Biosciences) at first diagnosed. These serum samples were drawn into sterile tubes and used immediately for analysis or frozen at −80 °C. The study was approved by the ethics committee of Nanjing Medical University, and all patients were given written informed consent before the study.

### Cells culture

Normal breast epithelial cells MCF-10A and human breast cancer cell line MCF-7, MDA-MB-231, and BT549 cells were purchased from the Cell Bank of the Chinese Academy of Sciences (Shanghai, China). Cell lines were cultured in Dulbecco’s modified Eagle’s medium (DMEM) high glucose (HyClone, USA) or RPMI1640 medium and supplemented with 10% fetal bovine serum (FBS) (Gibco, Australia), 100 μg/ml penicillin, and 100 U/ml streptomycin. All cells were cultured at 37 °C with 5% CO_2_.

### Isolation and characterization of exosomes

All cells were cultured when reached 50% confluency, and then wash with phosphate buffer solution (PBS) twice and incubated with DMEM containing exosomes-free FBS for 48 h. All exosomes of MCF-7 and MDA-MB-231 cells were harvested from supernatants and isolated by differential centrifugation and ultracentrifugation by an Avanti J-30I (Beckman Coulter, USA). Different cell culture supernatants were centrifuged at 300 × *g* for 15 min at 4 °C, then 16,500 × *g* for 30 min at 4 °C to remove redundant cells and cell debris. After that, all supernatants were filtrated through a 0.22-mm filter. Exosomes were pelleted by ultracentrifugation at 100,000 g for 2 h at 4 °C. The samples of serum from peripheral blood were centrifuged at 300 × *g* for 15 min and 16,500 × *g* for 30 min at 4 °C to remove redundant cells and cell debris. Then exosomes from serum were isolated by exoRNeasy Serum*/*Plasma Maxi Kit (Qiagen, Hilden, Germany) according to the manufacturer’s protocols. All samples of exosomes were resuspended in PBS and frozen at −80 °C or used immediately for analysis.

### Transmission electron microscopy

The isolated exosomes derived from MCF-7 and MDA-MB-231 cells were fixed with glutaraldehyde (2%) at 4 °C overnight. Then, all subjects were washed with PBS twice and precipitated in 1% OsO4 for 1 h at room temperature. The precipitates were dehydrated in ethyl alcohol, and embedded in epoxy resin. Pictures of exosomes derived from MCF-7 and MDA-MB-231 cells were acquired by Tecnai G2 Spirit Bio TWIN transmission electron microscope (FEI, USA).

### Profiling of circRNA from breast cells and their exosomes

TRIzol reagent (Invitrogen, MD, USA) was used for total RNA extracted from breast cancer cells and their exosomes. Spectrophotometrically 2000 (Thermo Fisher Scientific, USA) and an Agilent 2100 Bioanalyzer (Agilent Technologies, USA) were used to quantify total RNA. Moreover, all total RNA of breast cell lines and their exosomes was treated with Ribonuclease R (Rnase R) to remove linear RNAs before the construction of RNA-seq libraries. Strand-specific RNA libraries were constructed by using the VAHTS Total RNAseq (H*/*M*/*R) Library Prep Kit (Vazyme, China) according to the manufacturer’s protocols for Illumina. Bioanalyzer 4200 (Agilent, USA) was used to analyze purified first-strand cDNA of the libraries through PCR amplification. Finally, the cDNA was sequenced using an Illumina HiSeq X Ten system (Illumina, USA). All data can be found in previous articles published by our team by Dr. Zhong^17^.

### Identification of differentially expressed circRNAs from breast cells and their exosomes

All expression data were normalized with the ‘normalizeBetweenArrays’ algorithm by R-package ‘limma’[28]. Differential circRNAs expression between two groups were identified by ‘eBayes’ algorithm and moderated t-statistics by R-package ‘limma’[29]. The circRNAs with the *P*-value < 0.05 and |logFC(logFoldChange)| > 1 were selected as differential circRNAs.

The expression profiles of the top 39 differentially expressed circRNAs (both in cells and their exosomes) were shown in Additional file 1: Table S2. Sanger sequencing was used to identify the back splice junction of circPSMA1 by genewiz (Su Zhou, China). The primers of Sanger sequencing (circPSMA1-S-F/R) were shown in Additional file 1: Table S3.

### RNA extraction and quantitative real-time polymerase chain reaction (RT-qPCR)

Total RNA of exosomes was extracted through the Total Exosome RNA and Protein Isolation Kit (Invitrogen, USA). Total RNA of cell was extracted by RNA simple Total RNA kit (TIANGEN, Beijing, China) according to the manufacturer’s protocol. Nanodrop 2000 spectrophotometry (Thermo Scientific, USA) was used to measure the concentration and quality of the RNA by the UV absorbance at 260 and 280 nm. CircRNAs and miRNAs from cells or exosomes were reversed transcribed to synthesize cDNA according to the manufacturer’s instructions through PrimeScript™ RT reagent Kit (Takara, Japan) or Mir-X miRNA First-Strand Synthesis Kit (Takara, Japan) on an iCycler iQ system (Bio- Rad, USA). RT-qPCR was used to verify the accuracy levels of the differential circRNAs. RT-qPCR was performed by the LightCycler 480 SYBR Green I Master (Roche, Australia). The divergent primers of circRNA were designed for an exon sequence near the back-splice site of the corresponding circRNA by CircPrimer software (http://www.bioinf.com.cn/)^55^. The Ct values for each circRNA or miRNA were normalized to GAPDH or U6. All primers were shown in Additional file 1: Table S3. The relative expressions of circRNAs and miRNAs were calculated using the 2^−ΔΔCt^ method.

### The uptake of labeled exosomes by recipient cells

Following the manufacturer’s instructions, exosomes were labeled with a PKH26 Red Fluorescent Labeling Kit (Sigma-Aldrich, USA). GFP-labeled-MDA-MB-231 cells were incubated with the different amounts (10/50/100 µg) of PKH26-labeled exosomes derived from MDA-MB-231 cells. Recipient cells were incubated with the PKH26-labeled exosomes for 24 h. After co-cultured with the labeled exosomes, cells were washed in PBS and fixed with 4% paraformaldehyde for 15 min at room temperature in dark. Then cells were counterstained with Hoechst 33342 (1:1000 dilution; Thermo Fisher Scientific, USA), and subjected to an LSM510 confocal laser scanning microscope (Carl Zeiss, Germany).

### Fluorescence in situ hybridization (FISH)

The FISH assay used Fluorescent In Situ Hybridization Kit based on the manufacturer’s protocols (Gene-Pharma, China). The hybridization was performed with Cy3-labeled circPSMA1 probe (RIBOBIO, Guangzhou, China) and FAM-labeled miR-637 probe (5′-ACGCAGAGCCCGAAAGCCCCCAGT-3′; Gene-Pharma, China) and all samples were analyzed by confocal microscopy (Carl Zeiss, Germany).

### Plasmids construction, siRNA, miRNA mimics (inhibitors), and transfection

Full-length of circPSMA1 was subcloned into the pHB-circBasic vector (HANBIO, Shanghai, China) according to the manufacturer’s instructions. The circPSMA1 over-expressed plasmid was selected with penicillin and verified by sequencing. To knock down circPSMA1, three siRNAs that targeted the back-splice junction site of circPSMA1 were synthesized (RIBOBIO, Guangzhou, China). The efficiency examination was performed by qRT-PCR, siRNA-1 was recognized as the most effective one (Additional file 2: Fig. S1). All siRNA, mimics, and inhibitors were synthesized by RiboBio (Guangzhou, China). The sequences of siRNA, mimics, and inhibitors used are listed in Additional file 1: Table S4. We use electrotransfection to transfect cells with circPSMA1 over-expressed plasmid by a NEPA 21 electroporator (NEPA, Japan). The parameters of electrotransfection were described as follows: voltage, 120 V; pulse length, 5 ms; pulse interval, 5 ms; number of pulses, 2; decay rate, 10%. The sequence of circPSMA1 was subcloned into EF1a-GFP/Puro lentiviral vectors to construct circPSMA1 over-expressed vector in HEK293T cells for animal study (Gene-Pharma, China). The sequences of all constructs were verified by sequencing. MDA-MB-231 cells infected with lentivirus circPSMA1 over-expressed vector according to the manufacturer’s instructions and then selected with 1 μg/mL puromycin.

### Cell proliferation, cell cycle, and apoptosis assays

The proliferation activity of TNBC cells was stained by Cell-Light™ EdU DNA Cell Proliferation Kit (Ribobio, Guangzhou, China). Images were observed by Zeiss Axio Vert. A1 inverted fluorescent microscope (Carl Zeiss Microscopy GmbH, Germany). TNBC cells (BT-549, MDA-MD-231) were used to examine cloning capability by colony formation assays in 6-well plate. The cell cycle of TNBC cells and apoptotic assays were analyzed by flow cytometry (BD Biosciences, Franklin Lakes, NJ). TNBC cells were stained with PI for cell cycle analysis in accordance with the manufacturer’s protocols. The percentage of apoptotic cells were double stained with fluorescein isothiocyanate (FITC)-conjugated Annexin V and propidium iodide (PI).

### Wound healing and transwell assay

For wound healing assay, 3 × 10^5^ TNBC cells (BT-549, MDA-MD-231) were seeded into a 6-well plate and scratched with a 200 μL pipette tip in the middle of the wells at 24 h after transfection, then cultured in serum-free medium. Images were obtained using Zeiss Axio Vert. A1 inverted fluorescent microscope (Carl Zeiss Microscopy GmbH, Germany) at 0 and 24 h. Transwell assay was carried out in 24-well plate transwell inserts (Millipore, MA, USA). Five hundred microliters of DEME or RPMI1640 medium with 20% FBS were put into the bottom chamber and 2 × 10^4^ cells in 200 μL DEME or RPMI1640 medium without FBS were seeded into the upper chamber. After 24 to 48 h, we used 4% paraformaldehyde to fix the samples for 30 min and all inserts were stained by crystal violet. All pictures were observed by Zeiss Axio Vert. A1 inverted fluorescent microscope (Carl Zeiss Microscopy GmbH, Germany).

### MiRNA and mRNA prediction

CircMir software (http://www.bioinf.com.cn/?page_id=10/) was used to predict the certain miRNAs of the corresponding circRNAs^55^. The software depends on the online tools miRanda (http://www.microrna.org/microrna/getDownloads.do) and RNAhybrid-2.1.2 (https://bibiserv.cebitec.uni-bielefeld.de/rnahybrid/). Online tool TargetScan (http://www.targetscan.org) was also used to select the Predicted mRNAs. The signaling pathways of miRNAs were investigated by DIANA-miRPath v3.0 (http://snf-515788.vm.okeanos.grnet.gr/).

### Dual-luciferase reporter assay

The circPSMA1 or Akt1 3′UTR sequences containing wild-type or mutant miR-637 binding sites were synthesized and subcloned into luciferase reporter vector psiCHECK2 (Promega, Madison, WI, USA), termed circPSMA1-WT, circPSMA1-Mut, Akt1 3′UTR-WT, and Akt1 3′UTR-Mut, respectively. These plasmids were co-transfected with miR-637 mimics or control into cells to examine the relative luciferase activity using Dual-Luciferase Assay Kit in accordance with the manufacturer’s protocols (Promega, Madison, WI, USA), the relative luciferase activity was calculated by SpectraMax i3x (Molecular Devices, CA, USA).

### Data acquisition and processing

Transcriptome RNA-seq data (GSE65194) of 164 breast tissue samples and technical duplicates (55 TNBC, 39 Her2, 30 Luminal B, 29 Luminal A, 11 normal breast tissue samples) and their corresponding clinical data were acquired from the Gene Expression Omnibus (GEO) database (https://www.ncbi.nlm.nih.gov/geo/). The probe names of each gene were converted to gene symbols, moreover, when there are multiple probes corresponding to one gene, we define the expression level of the gene as the average value of several probes. Heatmaps of differential expressed genes (DEGs) were used R 4.02 “pheatmap” package with different breast cancer classification.

### TICs profile

We used CIBERSORT computational method to estimate the TIC abundance profile in all breast samples with quality filtering (*P* value < 0.05), and 103 samples were obtained for the following analysis. The correlation between different TICs was shown in a heatmap and the statistical significance of two TICs was calculated by Pearson correlation analyses. Violin plot showed the expression level of DEGs between TNBC samples (*n* = 55) and non-TNBC samples (*n* = 48).

### ESTIMATEScore, StromalScore, and ImmuneScore

ESTIMATE algorithm was used to estimate the immune or stromal component in Tumor microenvironment (TME) by R 4.02 “estimate” package. The values of ESTIMATEScore, StromalScore, and ImmuneScore were positive correlated to the ratio of immune-stromal, stromal and immune components in TME, respectively. Kruskal–Wallis rank sum test or Wilcoxon rank sum test was used to determine whether the above three scores were associated with certain clinicopathological features.

### Survival and tumor metastasis analysis

Hundred and thirty tumor samples with their overall survival (OS) time and the metastasis time were selected for the survival analysis by R 4.02 “survival” and “survminer” package. These samples were divided into two groups by the mean value of the score. The survival curve was plotted by Kaplan–Meier method, and the log rank was used as statistical significance test.

### DEGs between high and low ImmuneScore or StromalScore

ImmuneScore or StromalScore with corresponding gene expression were used to generate DEGs. The samples were divided into two groups by the median value of the score and calculated by R 4.02 “limma” package with the criteria of logFC > 0.8, *P* < 0.01, and false discovery rate (FDR) < 0.01. We used R 4.02 “pheatmap” package to produce heatmaps and R 4.02 “VennDiagram” package to show venn of 3744 up- and downregulated DEGs both in data regarding ImmuneScore or StromalScore.

### Gene ontology (GO) and Kyoto Encyclopedia of Genes and Genomes (KEGG) enrichment analysis

3744 DEGs were performed to acquired enriched cellular component (CC), molecular function (MF), biological process (BP), and pathway by R 4.02 “clusterProfiler”, “org.Hs.eg.db”, “enrichplot”, “ggplot2” package with the criteria of both *p* value < 0.05 and *q* value < 0.05.

### MiR-637 target gene and PI3K-Akt signaling pathway

PI3K-Akt signaling pathway was significantly enriched in KEGG Enrichment Analysis. Seventy-seven genes in PI3K-Akt signaling pathway and 5207 miR-637 target gene using TargetScan tool were shown in a venn diagram, and 23 genes were found in both data sets (AKT1, CCND1, CDKN1A, COL1A1, FOXO3, GRB2, IL2RB, IL7R, ITGA5, PTEN, THBS4, AKT2, ANGPT4, CHRM1, EGFR, FLT3LG, GNG4, LAMA4, LPAR3, NTRK2, PDGFB, PDPK1, PRKCA).

### Western blot analysis

Proteins from breast cancer cells were generated using a RIPA lysis buffer referring to the manufacturer’s instruction (Biouniquer Technology, China). Nanodrop 2000 spectrophotometry (Thermo Scientific, USA) was used to measured protein content. Same amounts of proteins were separated by electrophoresis using 10% sodium dodecyl sulfate polyacrylamide gels (SDS-PAGE) before transferring to a PVDF membrane (Bio-Rad, CA, USA). The membranes were blocked with 5% skimmed milk powder for 2 h at room temperature and then incubated with anti-Akt1 antibody (1:1000), anti-β-catenin antibody (1:500), anti-cyclin D1 antibody (1:500) (Cell Signaling Technology, USA) and anti-GAPDH antibody (1:5000, Proteintech, USA) at 4 °C overnight. After wash with TBST, all samples were incubated with secondary antibody (1:4000, Cell Signaling Technology, USA) for 1 h. Finally, enhanced chemiluminescence (ECL) plus kit (Millipore, America) was used for visualization and the images were analyzed by software Image J.

### Immunofluorescence (IF) and immunohistochemistry (IHC) assays

For IF assay, breast cancer cells were cultured to ~70% confluence, and then fixed with 4% paraformaldehyde for 30 min at room temperature. After washing with PBS, cells were permeabilized with 0.5% Triton X- 100 in PBS for 20 min on ice and then blocked with blocking buffer (1× PBS, 5% BSA, 0.3% Triton X-100) for 60 min at room temperature. Samples were incubated with anti-Akt1 antibody (1:200; Cell Signaling Technology, USA) for overnight at 4 °C. After wash with PBS, samples were incubated with Alexa Fluor 488 conjugated goat rabbit anti-IgG secondary antibody (1:250; Cell Signaling Technology, USA) for 1 h at room temperature in the dark. Finally, all samples were counterstained with Hoechst 33342 (1:1000 dilution; Invitrogen, USA) and observed by Zeiss Axio Vert. A1 inverted fluorescent microscope (Carl Zeiss Microscopy GmbH, Germany). For IHC assay, paraffin sections of tumors were incubated with primary antibodies: anti-Akt1 antibody (1:100), anti-β-catenin antibody (1:100), anti-cyclin D1 antibody (1:100), and anti-Bax antibody (1:100) (Cell Signaling Technology, USA) at 4 °C overnight and then incubated with the GTVision III Detection System/Mo&Rb Kit (Gene Tech Co., Ltd., Shanghai, PR China).

### Animal experiments

The animal studies were approved by the Animal Care and Use Committee of Nanjing Medical University (acceptance no.: IACUC 1903021) and complied with the guidelines of the National Institutes of Health. All the mice were randomly divided into two groups.2*10^6^ stably over-expressed circPSMA1 MDA-MB-231 cells (circ-PSMA1) and their control vectors (circ-NC) were gently injected into the mammary fat pad of female BALB/c nude mice (7 mice in each group) using an insulin syringe. All mice were housed in an air-filtered and pathogen-free condition. The length (*L*) and width (*W*) of tumor volume (*V*) were measured by exploiting vernier caliper once a week. The tumor volume was estimated by the formula *V* = (*LW*^2^)/2. After six weeks, all mice were euthanized and tumors along with livers and lungs were collected for further analysis. The metastatic nodules of livers and lungs were counted after H&E staining.

### Analysis of public databases

To evaluate the prognostic value of Akt1 and miR-637, we performed Kaplan–Meier survival analysis in TNBC patients with lymphatic metastasis from the Cancer Genome Atlas (TCGA) database (http://kmplot.com/analysis/). The auto select best values of Akt1 and miR-637 in TNBC were set to the cutoff values of the OS curves.

### Statistical analysis

Student’s *t*-test, one-way ANOVA, Kruskal–Wallis rank sum test or Wilcoxon rank sum test were used to compare differences between groups. Statistical significance was considered when *p*-value < 0.05. Statistical analysis was performed by SPSS 21.0 software and GraphPad Prism 8 (GraphPad Software Inc., CA, USA). All data were shown as mean ± standard deviation (SD).

## Supplementary information

Figure S1

Figure S2

Supplementary information
