# Supplementary material for: Tumor-derived exosomal circPSMA1 facilitates the tumorigenesis, metastasis, and migration in triple-negative breast cancer (TNBC) through miR-637/Akt1/β-catenin (cyclin D1) axis
Source: Cell Death Dis. 2021 Apr 28;12(5):420. doi: 10.1038/s41419-021-03680-1 (PMC8080849; doi:10.1038/s41419-021-03680-1)
Supplement: Supplementary file 3 — Supplementary information [file 41419_2021_3680_MOESM3_ESM.docx]

**Table S1 The clinical characteristics of 40 breast cancer patients.**

| Variables | | Cases (n=40) |
| --- | --- | --- |
| Age (years) | |  |
| ≤ 50 | | 18 |
| > 50 | | 22 |
| menopause | |  |
| no | | 21 |
| yes | | 19 |
| Tumor size (cm) | | |
| ≤ 2 | 26 | |
| > 2 | 14 | |
| TNM Staging |  | |
| I-II | 22 | |
| III-IV | 18 | |
| LN Infiltrated |  | |
| No | 24 | |
| Yes | 16 | |
| Histological grade | | |
| Well differentiated 6 | | |
| Moderately differentiated 14 | | |
| Poorly differentiated 20 | | |

TNM:TNM Classification of Malignant Tumors

**Table S2 The expression profiles of the top 39 differentially expressed circRNAs (both in cells and their exosomes).**

| **circRNA** | **231-exo** | **7-exo** | **231-cell** | **7-cell** | **Length** | **GeneSymbol** |
| --- | --- | --- | --- | --- | --- | --- |
| hsa_circ_0003312 | 58.23 | 86.71 | 305.66 | 105.45 | 329 | RELB |
| hsa_circ_0006047 | 98.71 | 65.91 | 481.74 | 277.72 | 360 | SNX25 |
| hsa_circ_0006456 | 8.25 | 2.15 | 53.15 | 6.36 | 6589 | SLC38A9 |
| hsa_circ_0011019 | 215.23 | 283.93 | 1931.21 | 1056.89 | 837 | ARID1A |
| hsa_circ_0011099 | 210.23 | 224.57 | 871.87 | 583.21 | 913 | RPA2 |
| hsa_circ_0012210 | 226.42 | 160.58 | 2987.68 | 991.33 | 423 | UROD |
| hsa_circ_0015719 | 44.42 | 49.73 | 1709.28 | 822.5 | 1595 | TPR |
| hsa_circ_0018105 | 5.53 | 2.9 | 198.54 | 83.48 | 2875 | KIF5B |
| hsa_circ_0018106 | 51.5 | 21.79 | 568.72 | 190.75 | 3113 | KIF5B |
| hsa_circ_0021335 | 18.81 | 2.48 | 2092.28 | 979.96 | 411 | PSMA1 |
| hsa_circ_0022514 | 549.5 | 614.75 | 1478.7 | 303.48 | 2753 | ZBTB3 |
| hsa_circ_0026763 | 82.78 | 202.78 | 1649.66 | 779.69 | 206 | ITGA5 |
| hsa_circ_0031188 | 107.05 | 176.63 | 1318.28 | 744.24 | 885 | CHD8 |
| hsa_circ_0038546 | 221.51 | 267.94 | 3956.47 | 1952.96 | 1569 | - |
| hsa_circ_0054222 | 51.94 | 38.22 | 260.54 | 154.44 | 396 | THUMPD2 |
| hsa_circ_0056460 | 181.55 | 126.68 | 936.83 | 536.27 | 726 | IMP4 |
| hsa_circ_0060757 | 275.81 | 208.82 | 10490.07 | 5432.03 | 1103 | CSE1L |
| hsa_circ_0062820 | 490.43 | 830.34 | 5100.55 | 2226.06 | 699 | MTMR3 |
| hsa_circ_0070337 | 18.84 | 8.63 | 328.08 | 114.62 | 687 | WDFY3 |
| hsa_circ_0087213 | 121.41 | 20.08 | 4489.81 | 1687.58 | 770 | ANXA1 |
| hsa_circ_0094728 | 17.2 | 2.3 | 394.54 | 43.67 | 6677 | BIRC2 |
| hsa_circ_0096303 | 15.4 | 4.34 | 763.58 | 226.85 | 391 | PPP6R3 |
| hsa_circ_0109036 | 33.08 | 32.65 | 85.59 | 23.9 | 6054 | - |
| hsa_circ_0109523 | 209.53 | 402.7 | 422.57 | 142.31 | 403 | ZNF829 |
| hsa_circ_0109619 | 282.99 | 411.38 | 3601.58 | 2076.37 | 4279 | SUPT5H |
| hsa_circ_0111209 | 1495.58 | 3101.17 | 13623 | 8401.99 | 514 | RC3H1 |
| hsa_circ_0111301 | 30.58 | 43.87 | 944.93 | 329.55 | 8847 | GNB1 |
| hsa_circ_0113456 | 15.22 | 9.53 | 46.48 | 30.22 | 482 | IPP |
| hsa_circ_0113532 | 51.87 | 82.86 | 127 | 46.18 | 9797 | EPS15 |
| hsa_circ_0115301 | 55.77 | 56.02 | 134.3 | 45.51 | 8417 | ZMYND8 |
| hsa_circ_0118180 | 51.75 | 16.67 | 1222.98 | 442.17 | 785 | SMC6 |
| hsa_circ_0119691 | 48.79 | 92.59 | 538.85 | 219.27 | 462 | ZNF512 |
| hsa_circ_0123044 | 42.82 | 33.89 | 437.66 | 82.52 | 619 | MAP3K13 |
| hsa_circ_0126565 | 46.13 | 25.07 | 2100.68 | 693.1 | 9007 | OCIAD1 |
| hsa_circ_0127793 | 132.1 | 207.28 | 626.94 | 74.19 | 4560 | ZNF608 |
| hsa_circ_0131114 | 34.26 | 33.63 | 153.61 | 67.27 | 4498 | CNKSR3 |
| hsa_circ_0131664 | 196.93 | 163.42 | 215.81 | 98.26 | 8013 | MAPK14 |
| hsa_circ_0140292 | 15.48 | 14.52 | 741.35 | 450.23 | 536 | USP9X |
| hsa_circ_0140364 | 10.63 | 17.83 | 88.95 | 15.12 | 316 | CCNB3 |

**Table S3 Sequences of all primers used in this study.**

| **Name** | **Sequence (5'to3')** |
| --- | --- |
| circPSMA1-forward | TGGTGTTGGTCTCCTTATTGC |
| circPSMA1-reverse | GGGCTCCAAACAGTGACATC |
| circPSMA1-S-forward | CAACACAACGATATGGCCGG |
| circPSMA1-S-reverse | TTGATGAATCCTGCCCTGGG |
| linear-PSMA1-forward | CAAACTCCCGCAGACTTCTC |
| linear-PSMA1-reverse | GACCAACTGTGGCTGAACCT |
| hsa-miR-323b-5p | AGGTTGTCCGTGGTGAGTTCGCA |
| hsa-miR-21-3p | CAACACCAGTCGATGGGCTGT |
| hsa-miR-132-3p | TAACAGTCTACAGCCATGGTCG |
| hsa-miR-193a-5p | TGGGTCTTTGCGGGCGAGATGA |
| hsa-miR-34b-5p | TAGGCAGTGTCATTAGCTGATTG |
| hsa-miR-505-3p | CGTCAACACTTGCTGGTTTCCT |
| hsa-miR-637 | ACTGGGGGCTTTCGGGCTCTGCGT |
| hsa-miR-874-3p | CTGCCCTGGCCCGAGGGACCGA |
| hsa-miR-1972 | TCAGGCCAGGCACAGTGGCTCA |
| hsa-miR-15a-3p | CAGGCCATATTGTGCTGCCTCA |
| has-miR-reverse | CAGTGCGTGTCGTGGAGT |
| U6-forward | CTCGCTTCGGCAGCACA |
| U6-reverse | AACGCTTCACGAATTTGCGT |
| Akt1-forward | CTGAGATTGTGTCAGCCCTGGA |
| Akt1-reverse | CACAGCCCGAAGTCTGTGATCTTA |
| GAPDH-forward | CGCTCTCTGCTCCTCCTGTTC |
| GAPDH-reverse | ATCCGTTGACTCCGACCTTCAC |

**Table S4 Sequences of siRNAs, mimics and inhibitors used in this study.**

| Name | Sequence |
| --- | --- |
| siRNA-1 | GCTGGTTATGATTTTCGAA |
| siRNA-2 | ATTGCTGGTTATGATTTTC |
| siRNA-3 | TGCTGGTTATGATTTTCGA |
| mimics-NC | UUUGUACUACACAAAAGUACUG, CAGUACUUUUGUGUAGUACAAA |
| Inhibitors-NC | CAGUACUUUUGUGUAGUACAAA |
| hsa-miR-323b-5p-mimic | AGGUUGUCCGUGGUGAGUUCGCA，UGCGAACUCACCACGGACAACCU |
| hsa-miR-21-3p-mimic | CAACACCAGUCGAUGGGCUGU，ACAGCCCAUCGACUGGUGUUG |
| hsa-miR-132-3p-mimic | UAACAGUCUACAGCCAUGGUCG，CGACCAUGGCUGUAGACUGUUA |
| hsa-miR-193a-5p-mimic | UGGGUCUUUGCGGGCGAGAUGA，UCAUCUCGCCCGCAAAGACCCA |
| hsa-miR-34b-5p-mimic | UAGGCAGUGUCAUUAGCUGAUUG，CAAUCAGCUAAUGACACUGCCUA |
| hsa-miR-505-3p-mimic | CGUCAACACUUGCUGGUUUCCU，AGGAAACCAGCAAGUGUUGACG |
| hsa-miR-637-mimic | ACUGGGGGCUUUCGGGCUCUGCGU,ACGCAGAGCCCGAAAGCCCCCAGU |
| hsa-miR-874-3p-mimic | CUGCCCUGGCCCGAGGGACCGA，UCGGUCCCUCGGGCCAGGGCAG |
| hsa-miR-1972-mimic | UCAGGCCAGGCACAGUGGCUCA，UGAGCCACUGUGCCUGGCCUGA |
| hsa-miR-15a-3p-mimic | CAGGCCAUAUUGUGCUGCCUCA，UGAGGCAGCACAAUAUGGCCUG |
| hsa-miR-637-inhibitor | ACGCAGAGCCCGAAAGCCCCCAGU |

**Table S5 The expression levels of Akt1 in various subtypes of breast cancer and normal tissues.**

| **Group** | **Mean Diff** | **95% CI of diff** | **Adjusted P Value** | **Significant** |
| --- | --- | --- | --- | --- |
| TNBC vs Healthy | 1.052 | 0.4220 to 1.682 | <0.0001 | **** |
| TNBC vs HER2 | -1.498 | -1.898 to -1.099 | <0.0001 | **** |
| TNBC vs Luminal A | 5.837 | 5.399 to 6.275 | <0.0001 | **** |
| TNBC vs Luminal B | 5.832 | 5.399 to 6.265 | <0.0001 | **** |
| Healthy vs HER2 | -2.550 | -3.202 to -1.899 | <0.0001 | **** |
| Healthy vs Luminal A | 4.785 | 4.110 to 5.461 | <0.0001 | **** |
| Healthy vs Luminal B | 4.780 | 4.107 to 5.452 | <0.0001 | **** |
| HER2 vs Luminal A | 7.336 | 6.868 to 7.803 | <0.0001 | **** |
| HER2 vs Luminal B | 7.330 | 6.867 to 7.794 | <0.0001 | **** |
| Luminal A vs Luminal B | -0.005 | -0.5022 to 0.4915 | >0.9999 | ns |

**Table S6 The expression levels of CCND1 (cyclinD1) in various subtypes of breast cancer and normal tissues.**

| **Group** | **Mean Diff** | **95.00% CI of diff** | **Adjusted P Value** | **Significant** |
| --- | --- | --- | --- | --- |
| TNBC vs Healthy | -0.2205 | -1.457 to 1.016 | 0.988 | ns |
| TNBC vs HER2 | -0.7506 | -1.534 to 0.03322 | 0.0676 | ns |
| TNBC vs Luminal A | 4.665 | 3.806 to 5.524 | <0.0001 | **** |
| TNBC vs Luminal B | 4.54 | 3.690 to 5.389 | <0.0001 | **** |
| Healthy vs HER2 | -0.5301 | -1.808 to 0.7481 | 0.7827 | ns |
| Healthy vs Luminal A | 4.886 | 3.560 to 6.211 | <0.0001 | **** |
| Healthy vs Luminal B | 4.76 | 3.440 to 6.080 | <0.0001 | **** |
| HER2 vs Luminal A | 5.416 | 4.498 to 6.334 | <0.0001 | **** |
| HER2 vs Luminal B | 5.29 | 4.381 to 6.199 | <0.0001 | **** |
| Luminal A vs Luminal B | -0.1256 | -1.101 to 0.8495 | 0.9966 | ns |
